# Role of inflammatory factors in prediction of Gleason score and its upgrading in localized prostate cancer patients after radical prostatectomy

**DOI:** 10.3389/fonc.2022.1079622

**Published:** 2023-01-12

**Authors:** Shuo Wang, Yongpeng Ji, Jinchao Ma, Peng Du, Yudong Cao, Xiao Yang, Ziyi Yu, Yong Yang

**Affiliations:** Key laboratory of Carcinogenesis and Translational Research (Mninistry of Education), Urological department, Peking University Cancer Hospital & Institute, Beijing, China

**Keywords:** prostate cancer, neutrophil to lymphocyte ratio, gleason score upgrading, systemic immune-inflammation index, inflammatory markers

## Abstract

**Purpose:**

To investigate the role of inflammatory factors including systemic immune-inflammation index (SII) and neutrophil to lymphocyte ratio (NLR) in predicting Gleason Score (GS) and Gleason Score upgrading (GSU) in localized prostate cancer (PCa) after radical prostatectomy (RP).

**Methods:**

The data of 297 patients who underwent prostate biopsy and RP in our center from January 2014 to March 2020 were retrospectively analyzed. Preoperative clinical characteristics including age, values of tPSA, total prostate volume (TPV), f/t PSA ratio, body mass index (BMI), biopsy GS and inflammatory factors including SII, NLR, lymphocyte to monocyte (LMR), neutrophil ratio (NR), platelet to lymphocyte ratio (PLR), lymphocyte ratio (LR), mean platelet volume (MPV) and red cell distribution (RDW) as well as pathological T (pT) stage were collected and compared according to the grades of RP GS (GS ≤ 6 and GS≥7), respectively. ROC curve analysis was used to confirm the discriminative ability of inflammatory factors including SII, NLR and their combination with tPSA for predicting GS and GSU. By using univariate and multivariate logistic regression analysis, the association between significant inflammatory markers and grades of GS were evaluated.

**Results:**

Patients enrolled were divided into low (GS ≤ 6) and high (GS≥7) groups by the grades of GS. The median values of clinical factors were 66.08 ± 6.04 years for age, 36.62 ± 23.15 mL for TPV, 26.16 ± 33.59 ng/mL for tPSA and 0.15 ± 0.25 for f/t PSA ratio, 22.34 ± 3.14 kg/m^2^ for BMI, 15 (5.1%) were pT1, 116 (39.1%) were pT2 and 166 (55.9%) were pT3. According to the student’s t test, patients in high GS group had a greater proportion of patients with pT3 (P<0.001), and higher NLR (P=0.04), SII (P=0.037) and tPSA (P=0.015) compared with low GS group, the distribution of age, TPV, f/t PSA ratio, BMI, LMR, NR, PLR, LR, MPV and RDW did not show any significantly statistical differences. The AUC for SII, NLR and tPSA was 0.732 (P=0.007), 0.649 (P=0.045) and 0.711 (P=0.015), with threshold values of 51l.08, 2.3 and 10.31ng/mL, respectively. According to the multivariable logistic regression models, NLR ≥ 2.3 (OR, 2.463; 95% CI, 0.679-10.469, P=0.042), SII ≥ 511.08 (OR, 3.519; 95% CI 0.891-12.488; P=0.003) and tPSA ≥ 10.31 ng/mL (OR, 4.146; 95% CI, 1.12-15.35; P=0.033) were all independent risk factors associated with higher GS. The AUC for combination of SII, NLR with tPSA was 0.758 (P=0.003) and 0.756 (P=0.003), respectively. GSU was observed in a total of 48 patients with GS ≤ 6 (55.17%). Then patients were divided into 2 groups (high and low) according to the threshold value of SII, NLR, tPSA, SII+tPSA and NLR+tPSA, respectively, when the GSU rates were compared with regard to these factors, GSU rate in high level group was significantly higher than that in low level group, P=0.001, 0.044, 0.017, <0.001 and <0.001, respectively.

**Conclusion:**

High SII, NLR and tPSA were associated with higher GS and higher GSU rate. SII was likely to be a more favorable biomarker for it had the largest AUC area compared with tPSA and NLR; the combination of SII or NLR with tPSA had greater values for predicting GS and GSU compared with NLR, SII or tPSA alone, since the AUC area of combination was much higher. SII, NLR were all useful inflammatory biomarkers for predicting GS and detecting GSU among localized PCa patients with biopsy GS ≤ 6.

## Introduction

Prostate cancer (PCa) is the second most common malignant tumors and fifth leading cause of malignant tumor death in male around the world (1). The most usually used method for diagnosis of PCa was prostate biopsy. However, the prostate biopsy is not accurate enough for judging the real Gleason Socre (GS), even with continuous progress of biopsy, differences still existed between biopsy and pathology after radical prostatectomy (RP) (2), which may be due to over time of the disease progression or insufficiency of basic biopsy (3). The GS has great values in the diagnosis, grading, therapeutic and prognosis among patients with PCa, the inaccurate of GS may affect the therapeutic strategy and prognosis of the patients. Therefore, some methods are needed for predicting the grade and post-operative changes in GS to guide the clinical decision making.

Recently, it has been proved that inflammation plays a crucial role in the occurrence and progression of malignant tumors. Several hemorrhagic based inflammatory markers including neutrophil ratio (NR), neutrophil to lymphocyte (NLR), platelet to lymphocyte ratio (PLR) have been investigated to be closely associated with the therapeutic efficiency, prognostic, pathological features and biochemical recurrence (BCR) among patients with PCa (4, 5). As one of the most important cancer related systemic inflammatory makers, NLR has been demonstrated could predict PCa and Gleason Score upgrading (GSU) in men underwent prostate needle biopsy (6, 7). Rulando et al. demonstrated that high NLR was related with higher GS and higher progression rate (8). Although these studies have explored the role of NLR in predicting GS and GSU, limited to the involved factors and sample size, conclusions drawn from these studies need to be further investigated (9, 10). In addition, systemic immune inflammation index (SII), a novel inflammatory index based on neutrophil, lymphocyte and platelet counts, has recently emerged as a more powerful biomarker predicting the occurrence and progression in various malignant tumors (11–13). In terms of PCa, in 2016, it was firstly investigated and was considered as a valuable marker predicting prognosis of metastatic castration resistance prostate cancer (mCRPC) (14). However, until now no data have been reported on the predictive values of SII on GS and GSU in localized PCa.

The aim of this retrospective study was to evaluate the values of preoperative SII and NLR in predicting grades of GS, and assessed their clinical usefulness in detecting the consistency of the GS between prostate biopsy and RP.

## Material and methods

Between January 2014 and March 2020, males with localized PCa who fulfill the inclusion criteria and underwent prostate biopsy and RP in our institution were all included in this study. The study was approved by the medical ethics review committee of Peking University Cancer Hospital & Institute (protocol code 2020KT30). Among these patients, no one received neo-adjuvant therapy before RP. Preoperative clinical characteristics including serum tPSA value, age, f/t PSA ratio, body mass index (BMI), total prostate volume (TPV), biopsy GS and complete blood counts (CBCs) based parameters including SII, NLR, NR, lymphocyte to monocyte (LMR), PLR, lymphocyte ratio (LR), red cell distribution (RDW) and mean platelet volume (MPV) as well as pathological T (pT) stage were collected and compared according to the grades of RP GS (GS ≤ 6 and GS≥7), respectively. Data of risk factors related to the grade of RP GS including SII, NLR and tPSA were collected and analyzed to detect their associations with the real GS. The pre-operative hematologic workup was performed approximately 1-2 days before RP. Patients with history of autoimmune or inflammatory disease, any surgical intervention within 1 month, acute or chronic infections and malignant tumors in other tissue or organs which may modify the levels of CBC based parameters were excluded from analysis.

### Procedure

In this cohort, all patients enrolled were with PSA > 4ng/mL, MRI was performed before biopsy, then 13-core trans-rectal prostatic biopsy guiding by ultrasound was performed in all patients. In accordance with our institutional policy, a minimum 5 weeks of wait time was needed for surgery from the most recent prostate biopsy date. Pre-operative CBC and tPSA value were performed as part of the routine assessment testing 1-2 days before RP and at least 4 weeks after prostate biopsy to minimize the effect of intervention. PSMA PET-CT was performed before RP to confirm no distant tissues, organs or bone metastasis. According to technique of Walsh et al. (15), extra-fascial RP was performed by sophisticated senior urologists in our center. Biopsy specimens and RP gross specimen pathological examination and diagnosis were jointly finished by the senior pathologists. The GS were graded according to the 2014 International Society of Urological Pathology (ISUP) Consensus Conference on Gleason Grading of Prostate Carcinoma (16).

### Variables

The dimension of the prostate was measured by pre-surgery MRI and RP specimen separately, and the volume was calculated with the modified ellipsoid formulation in cm^3^ (0.523 x [length × height × width]), both biopsy and RP GS were recorded and patients enrolled were staged according to the 2010 American Joint Committee on Cancer system (AJCC, pathological stage T1-T4) (17). Tumors were stratified into 2 groups according to RP GS (GS ≤ 6 and GS≥7). BMI=weight (kg)/height (m)^2^. Hematological parameters (LR, NR, MPV and RDW) were evaluated by using peripheral blood samples, and NLR, SII, PLR, LMR were calculated by the numbers of blood cell counts based systemic inflammatory markers. NLR and SII were calculated separately: NLR = neutrophil count/lymphocyte count, SII = neutrophil count × platelet count/lymphocyte count, SII has been presented as a combination of PLR and NLR (11, 18). Among patients with biopsy GS ≤ 6, the really GS was greater than 6 according to the RP specimen was defined as GSU.

### Statistical analysis

Shapiro-Wilk test were used for the measurement data analysis presented as Mean ± SD. Clinicopathological characteristics were compared between groups by independent t test for continuous variables, Chi-square tests for categorical variables. Receiver operating characteristic curve (ROC) was used to determine the threshold value of risk factors including NLR, SII and tPSA for predicting GS. Youden’s index was used calculating the specificity and sensitivity levels which was defined as YI_(C)_=max c [Se_(C)_+SP_(C)_-1]. Univariable and multivariable logistic regression analysis were performed to identify predicting factors for GS grading, which were all compared with reference group (Ref). In order to increase the sensitivity of detecting GS≥7 PCa from biopsy-based GS ≤ 6 PCa, we defined combination of tPSA with NLR (tPSA+NLR), tPSA with SII (tPSA+SII) was positive when either SII, NLR or tPSA indicated GS≥7 above cut-off values. The IBM SPSS software with version 20 was used to run the analysis. Two sided P value < 0.05 was considered statistically meaningful differences.

## Result

### Clinicopathologic characteristics of the patients

A total of 297 localized PCa patients who underwent RP (293 with laparoscopic and 4 with open) were enrolled into the cohort, all patients have underwent 13-core trans-rectal prostatic biopsy guiding by ultrasound and with pathology confirmed prostate adenocarcinoma before RP. The median values of clinical factors were shown in **Table 1**. On the basis of biopsy GS, there were 87 (29.29%) patients had histological GS ≤ 6 (3 with GS=5, 84 with GS=6) and 210 (70.71%) had GS≥7 (126 with GS=7, 84 with GS≥8); on the basis of RP pathological review, there were 39 (13.13%) patients had histological GS ≤ 6 (2 with GS=5, 37 with GS=6) and 258 (86.87%) had GS≥7 (132 with GS=7, 126 with GS≥8) respectively.

**Table 1:** Comparison of clinicopathologic features between study groups according to grades of RP GS.

Variables	All patients	RP GS ≤ 6	RP GS≥7	P value
NLR (%)	3.05 ± 1.72	2.29 ± 0.44	3.16 ± 1.81	0.04
SII (%)	607.38 ± 351.68	418.61 ± 117.91	636.25 ± 366.71	0.037
tPSA (ng/mL)	26.16 ± 33.59	13.27 ± 11.66	28.00 ± 35.30	0.015
Age (years)	66.08 ± 6.04	65.77 ± 6.19	66.13 ± 6.05	0.843
pT (n, %)	297	39	258	<0.001
pT1	15 (5.1)	12 (30.8)	3 (1.2)	
pT2	116 (39.1)	26 (66.7)	90 (34.9)	
pT3	166 (55.9)	1 (2.6)	165 (64.0)	
TPV (mL)				
MRI	35.59 ± 25.11	34.52 ± 15.16	36.12 ± 17.12	0.459
Specimen	36.62 ± 23.15	33.79 ± 11.14	36.96 ± 24.24	0.685
f/t PSA ratio	0.15 ± 0.25	0.16 ± 0.11	0.15 ± 0.26	0.901
LMR (%)	4.26 ± 1.91	4.59 ± 1.94	4.21 ± 1.91	0.51
NR (%)	65.18 ± 9.08	64.96 ± 5.85	65.21 ± 9.5	0.928
PLR (%)	146.31 ± 52.83	145.19 ± 42.21	146.48 ± 54.48	0.935
LR (%)	25.86 ± 8.09	26.92 ± 4.81	25.70 ± 8.49	0.615
MPV (10^3^/μL)	9.81 ± 1.10	9.94 ± 0.89	9.80 ± 1.14	0.665
RDW (%)	12.84 ± 0.78	12.76 ± 0.55	12.86 ± 0.81	0.686
BMI (kg/m^2^)	22.34 ± 3.14	24.58 ± 3.96	23.96 ± 4.18	0.67

Variables were compared to find the significant differences between patients with GS ≤ 6 and GS≥7. RP, radical prostatectomy; GS, Gleason Score; NLR, neutrophil to lymphocyte ratio; SII, systemic immune-inflammation index; tPSA, total prostate cancer specific antigen; pT, pathological T stage; TPV, total prostate volume; LMR, lymphocyte to monocyte ratio; NR, neutrophil ratio; PLR, platelet to lymphocyte ratio; LR, lymphocyte ratio; MPV, mean platelet volume; RDW, red cell distribution; BMI, body mass index.

### Analysis of clinical and CBC based parameters

By the grades of RP GS, our study cohort were divided into low (GS ≤ 6) and high (GS≥7) groups. Both groups had similar baseline clinical and CBC based parameters including age, f/t PSA ratio, TPV, LMR, NR, PLR, LR, MPV and RDW by Student’s *t* test, except that high GS group had a greater proportion of pT3 stage (P<0.001), and higher NLR (P=0.04), SII (P=0.037) and tPSA (P=0.015) compared with low GS group (**Table 1**).

**Figure 1:**
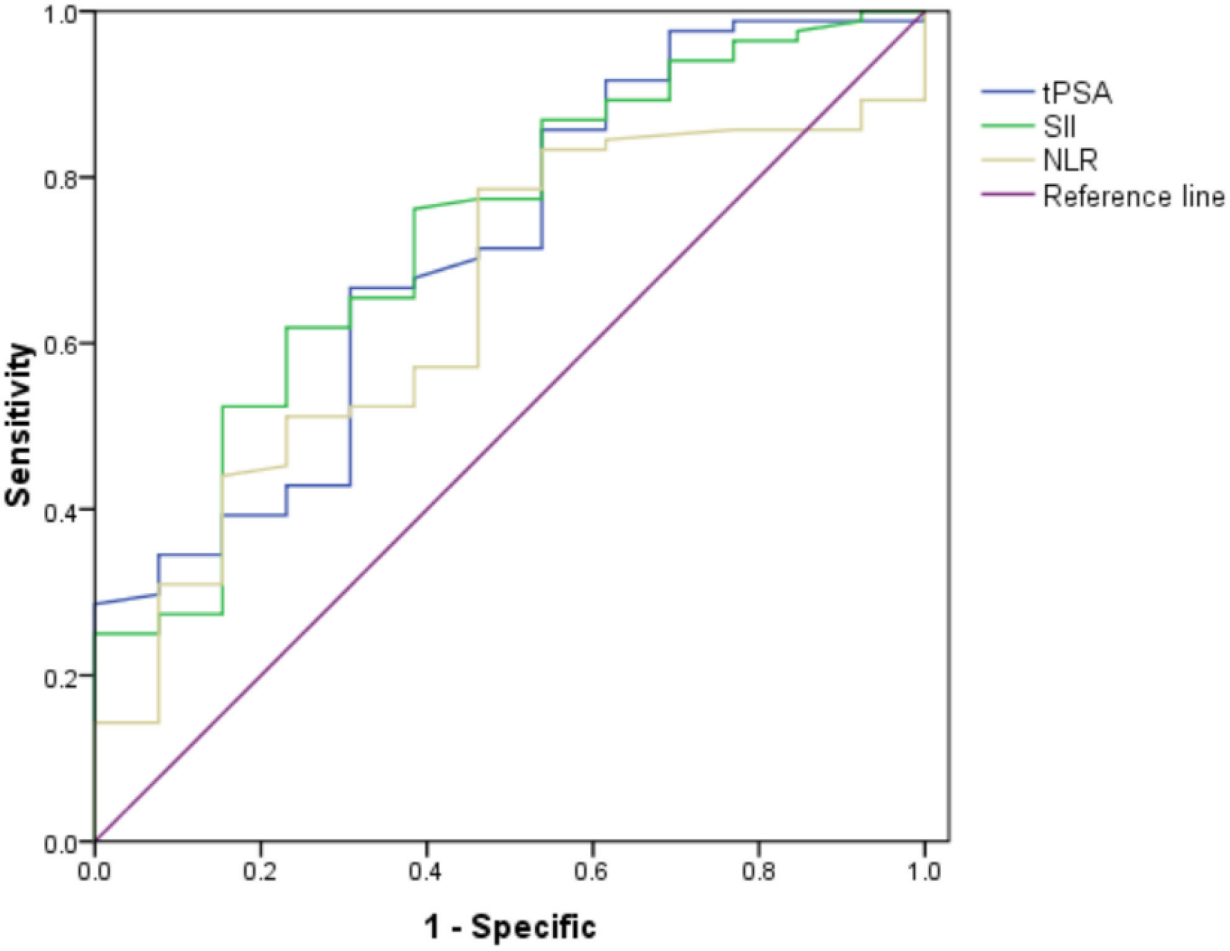
Role of SII, NLR and tPSA in predicting grades of real GS after RP by ROC curve analysis. The AUC for SII, NLR and tPSA was 0.732, 0.649 and 0.711, with P value 0.007, 0.045 and 0.015, respectively. SII got the largest AUC compared with NLR and tPSA.

The ROC curve for SII, NLR and tPSA was plotted in predicting higher GS (**Table 2** and **Figure 1**). In patients with GS≥7, AUC for SII was 0.732 with the P value of 0.007, threshold value of 511.08, sensitivity of 62.4% and specificity of 76.9%; AUC for NLR was 0.649 with the P value of 0.045, threshold value of 2.3, sensitivity of 78.8% and specificity of 53.8%; AUC for tPSA was 0.711 with the P value of 0.015, threshold valued of 10.31, sensitivity of 66.7% and specificity of 69.2%. Together a comparable value was found between SII and tPSA, SII exhibited better potential as an adjuvant biomarker predicting the grades of GS compared with NLR.

**Table 2:** Cut-off, AUC, sensitivity and specificity values of NLR and SII for predicting grades of GS.

Variables	AUC	cut-off	Sensitivity	Specificity	95% CI	P value
SII	0.732	≥511.08	62.4%	76.9%	0.592-0.878	0.007
NLR	0.649	≥2.3	78.8%	53.8%	0.508-0.798	0.045
tPSA	0.711	≥10.31	66.7%	69.2%	0.561-0.861	0.015

SII has the largest AUC with 0.732 compared with NLR, tPSA. AUC, area under the curve; NLR, neutrophil to lymphocyte ratio; SII, systemic immune-inflammation index; tPSA, total prostate cancer specific antigen; GS, Gleason Score.

Then patients were divided into 2 groups according to the threshold values of SII, NLR and tPSA, univariable and multivariable logistic regression models were used to make an assessment of the association between risk factors and GS. By using univariable analysis, NLR ≥ 2.3 (OR, 12.2; 95% CI, 4.902-30.361, P=0.007), SII ≥ 511.08 (OR, 16.667; 95% CI, 5.199-53.434, P<0.001) and tPSA ≥ 10.31 ng/mL (OR, 14.25; 95% CI, 5.171-39.273, P<0.001) were risk factors associated with higher GS (**Table 3**); In multivariable analysis (4 variables were included, NLR, SII, tPSA ans pT stage), NLR ≥ 2.3 (OR, 2.463; 95% CI, 0.679-10.469, P=0.042), SII ≥ 511.08 (OR, 3.519; 95% CI 0.891-12.488; P=0.003) and tPSA ≥ 10.31 ng/mL (OR, 4.146; 95% CI, 1.12-15.35; P=0.033) were all independent risk factors predicting higher GS (**Table 3**).

**Table 3:** Univariable and Multivariable analyses for predicting grades of GS.

	Univariable analysis RP GS ≤ 6 vs RP GS≥7			Multivariable analysis RP GS ≤ 6 vs RP GS≥7		
	OR	95% CI	P value	OR	95% CI	P value
NLR						
<2.3	Ref (1)	Ref (1)		Ref (1)	Ref (1)	
≥2.3	12.2	4.902-30.361	0.007	2.463	0.679-10.469	0.042
SII						
<511.08	Ref (1)	Ref (1)		Ref (1)	Ref (1)	
≥511.08	16.667	5.199-53.434	<0.001	3.519	0.891-12.488	0.003
tPSA (ng/mL)						
<10.31	Ref (1)	Ref (1)		Ref (1)	Ref (1)	
≥10.31	14.25	5.171-39.273	<0.001	4.146	1.12-15.35	0.033

NLR, SII and tPSA were all independent factors related with the grades of GS. NLR, neutrophil to lymphocyte ratio; RP, radical prostatectomy; SII, systemic immune-inflammation index; tPSA, total prostate cancer specific antigen; GS, Gleason Score; Ref, reference.

### ROC curve analysis of SII, NLR combined with tPSA for predicting GS

**Figure 2:**
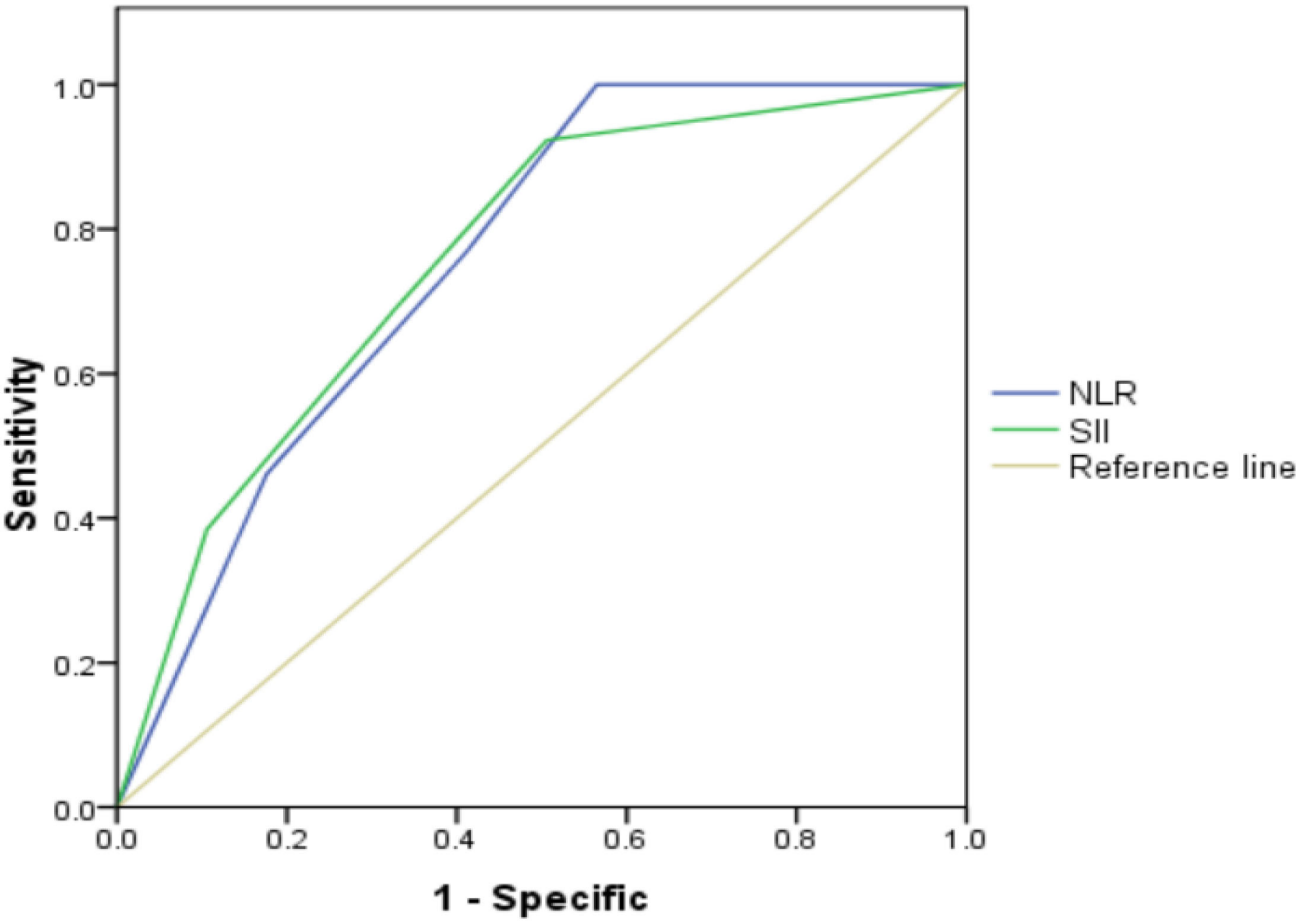
Role of SII, NLR combined with tPSA in predicting grades of real GS after RP by ROC curve analysis. The AUC for SII + tPSA, NLR + tPSA was 0.758 and 0.756, with P value 0.003 and 0.003, respectively. SII and NLR showed comparable values in predicting grades of real GS when combined with tPSA.

According to the threshold values of SII, NLR and tPSA, patients were classified and assessed by the combination of tPSA with SII, tPSA with NLR. The results revealed that AUC for tPSA + SII was 0.758 with the P value of 0.003, sensitivity of 46.2% and specificity of 82.4%; AUC for tPSA + NLR was 0.756 with the P value of 0.003, sensitivity of 38.5% and specificity of 89.4% (**Table 4** and **Figure 2**). SII and NLR showed comparable values in predicting higher GS when they combined with tPSA.

**Table 4:** AUC sensitivity and specific values of SII, NLR combined with tPSA for predicting grades of GS.

Variables	AUC	Sensitivity	Specificity	95% CI	P value
SII+tPSA	0.758	46.2%	82.4%	0.643-0.869	0.003
NLR+tPSA	0.756	38.5%	89.4%	0.629-0.887	0.003

NLR, neutrophil to lymphocyte ratio; SII, systemic immune-inflammation index; tPSA, total prostate cancer specific antigen; GS, Gleason Score; AUC, area under the curve.

### GSU rates with regard to NLR, SII, NLR + tPSA and SII + tPSA

GSU was observed in a total of 48 (55.17%) patients with GS ≤ 6. When the groups were compared with regard to NLR, SII and tPSA, there were 17(44.7%) and 31(63.3%) patients who had GSU in the NLR<2.3 and NLR≥2.3 groups, P=0.044; 20 (40%) and 28 (75.7%) patients who had GSU in the SII<511.08 and SII≥511.08 groups, P=0.001; 21 (43.8%) and 27 (69.2%) patients who had GSU in the tPSA <10.31 and tPSA≥10.31 groups, P=0.017, respectively (**Table 5**).

**Table 5:** Comparison of GSU rates between groups with regard of SII, NLR and tPSA.

Variables	SII<511.08 n=50	SII≥511.08 n=37	P value	NLR<2.3 n=38	NLR≥2.3 n=49	P value	tPSA<10.31, n=48	tPSA≥10.31, n=39	P value
GSU, n (%)			0.001			0.044			0.017
Yes	20 (40%)	28(75.7%)		17(44.7%)	31(63.3%)		21(43.8%)	27(69.2%)	
No	30 (60%)	9(24.3%)		21(55.3%)	18(36.7%)		27(56.2%)	12(30.8%)	

Rate of GSU was calculated and compared, it was much higher in patients with values of SII, NLR or tPSA above the cut-off line. GSU, Gleason Score upgrading; NLR, neutrophil to lymphocyte ratio; SII, systemic immune-inflammation index; tPSA, total prostate cancer specific antigen.

When the groups were compared with regard to NLR + tPSA and SII + tPSA, 2(5.6%) and 46(90.2%) patients were observed with GSU among groups divided by NLR + tPSA, P<0.001; 13(28.9%) and 35 (83.3%) patients were observed with GSU among groups divided by SII + tPSA, P<0.001 (**Table 6**).

**Table 6:** Comparison of GSU rates between groups with regard of SII+tPSA, NLR+tPSA.

Variables	SII+tPSA negative, n=45	SII+tPSA positive, n=42	P value	NLR+tPSA negative, n=36	NLR+tPSA positive, n=51	P value
GSU, n (%)			<0.001			<0.001
Yes 48 (55.17%)	13 (28.9%)	35 (83.3%)		2 (5.6%)	46 (90.2%)	
No 39 (44.83%)	32 (71.1%)	7 (16.7%)		34 (94.4%)	5 (9.8%)	

Rate of GSU was calculated and compared, it was much higher in patients with positive result of SII+tPSA or NLR+tPSA. NLR, neutrophil to lymphocyte ratio; SII, systemic immune-inflammation index; tPSA, total prostate cancer specific antigen; GSU, Gleason Score upgrading; SII+tPSA/NLR+tPSA negative means both SII, NLR and tPSA must under the cut-off values; NLR+tPSA/SII+tPSA positive means either NLR, SII or tPSA above the cut-off values.

## Discussion

GS is one of the most important indicators to evaluate the biological features of PCa, it is closely related to the BCR and positive surgical margins in localized PCa after RP (19, 20); It also associated with poorer therapy efficiency and prognostic in metastatic PCa patients (21). In addition, GS can help to guide the therapeutic modalities to choose, so accurately evaluated the real GS is the key to treatment and prognostic. The mostly usually used method assessing GS is prostate biopsy. However, the GS of prostate biopsy has an inherent sampling error and often differs from the GS of RP specimen (22). It is reported that the GS of biopsy differs as much as 60% to 70% from the GS of RP specimen (23, 24). GS differs between biopsy and RP specimen may result in delay of definitive treatment and mis-assessment of patient outcomes. Therefore, more applicable and available biochemical or biological markers are needed to get more accurate results.

tPSA is one potential predictor, it has been reported could correctly identify 60% GS≥7 and 80.3% GS ≤ 6 PCa with a cut-off value of 14.09ng/mL (24), in our study, we got the same conclusion that tPSA was positively associated with grades of GS and GSU rate with a cut-off value of 10.31ng/mL. In addition, some studies combined free and total PSA with [–2] pro-PSA, as well as four kallikrein protein biomarkers including tPSA, fPSA, intact PSA and human kallikrein related peptidase 2 to predict the GSU (25), but these tests are expensive and hard to be applied widely. TPV is another potential predictor (26), but according to our study, when we assessed the TPV by MRI and RP specimen respectively, we found there seemed no differences of TPV between GS ≤ 6 and GS≥7 group, then we further analyzed its relationship with grades of GS and GSU by ROC curve, the AUC was really low (AUC <60, P>0.05), so in our opinion it was not a candidate for predicting grades of GS and GSU. However, when we compared the TPV calculated by MRI with actual TPV of specimen, there do a strong correlation between them (Mean=-4.5122, SD=11.25; P<0.001), demonstrating that MRI is an adequate method in clinical practice for estimating the real TPV as reported by Matteo Massanova, et al. (27) In recent years, many studies have detected the mechanism of inflammatory incidence in tumors, they concluded that inflammatory factors may play a key role regulating the progression of disease by stimulating or suppressing tumor cells (28). NLR and SII are the most important inflammatory indexes, they can precisely reflect the immune-inflammatory status of the body. High NLR has been demonstrated to be associated with more aggressive and poorer response to treatment in metastatic PCa patients (29). Another study investigated the association between NLR and cancer specific survival time (CSS) in localized PCa, found there was a negative relationship between NLR and CSS (30). In our previous study, we had revealed that inflammatory factors including NLR, NR and SII were all related to the occurrence and BCR in localized PCa after RP (31, 32). More recently, Gokce et al. revealed that NLR was positively associated with GSU and BCR, the GSU rate was 25.9% and 39.6% regarding to NLR with cut-off value of 2.5 (7), which was consist with the result in this cohort. Another study published in 2017 demonstrated that high NLR was significantly related with GS≥7and it could be considered as a potential predictor for discriminating GS≥7 from biopsy-based GS ≤ 6 PCa (24). All these studies above revealed that NLR might be a potential marker to predict the pathological characteristics in patients with PCa. In our study, we concluded that NLR was positively correlated with the higher GS and GSU rate with the cut-off value of 2.3, which was consistent with the results of studies reported before.

Recently, beside NLR, a novel biomarker - SII which combines components of NLR and PLR, has shown promising values reflecting the systemic inflammatory responses which is more comprehensively than other inflammatory indexes. High SII suggested a weak adaptive immune response and an elevated non-specific inflammatory status in patients, which might promote the occurrence and development of tumors (33, 34). Several studies have investigated the association between SII and PCa. Man et al. explored the prognostic value of pretreatment SII in mCRPC patients treated with first-line docetaxel, concluded that high SII was associated with the poor outcomes in mCRPC patients after first-line docetaxel therapy (34). Rajwa et al. demonstrated that there was a strong correlation between high preoperative SII and adverse pathological features and high BCR with cut-off value of 620 in localized PCa patients conducted RP (35). Another retrospective study enrolled 230 mCRPC patients treated with Abiraterone, detecting the predicting role of SII for overall survival (OS), concluded that high level of SII (≥535) was associated with shorter OS in these patients (14). However, there is, to data, no data on the predictive values of SII on the grades of GS or GSU in localized PCa. In our study, we investigated the association of NLR, SII and tPSA with grades of GS and GSU in localized PCa patients, to the best of our knowledge, this is the first study investigating the relationship between SII and grades of GS, GSU, and the results indicated that high SII was related to the higher GS and higher GSU rate as well as NLR, they all could represent as the novel and reliable predictive markers for GS in localized PCa, but SII seemed more favorable for it had the largest AUC area of 0.732 compared with NLR of 0.649, tPSA of 0.711. Meanwhile, we combined SII, NLR with tPSA to evaluate their role in predicting GS and GSU, the combination seemed have obvious advantages compared with SII, NLR or tPSA alone since the AUC areas of combination (SII + tPSA of 0.758, NLR + tPSA of 0.756) were much higher, especially when compared with NLR of 0.649 alone. Therefore, the inflammatory factors has great values in clinic, it was easily available and could identify the real GS in patients underwent biopsy, thus it could predict the patients’ prognosis and make fully preparation for the following therapy strategies before RP pathology, but since the AUC for all the inflammatory markers were lower than 0.8, and the sensitivity and specificity were not very high, especially for NLR or SII alone, the inflammatory markers might be used as part of predictive evaluation but not a predictive tool.

Several limitations existed in this cohort. First and foremost are the limitations inherent to the retrospective data collection; Second, the high rate of patients with RP GS≥7 may lead to a potential bias of evaluation; Third, one single time point was used for measuring the biomarkers and although we have excluded patients with infection, inflammatory disease and so on, but there are too many factors that will influence the CBC based results, leading to the inaccurate of the data collected, it can be strengthened by collecting blood samples at different pre-operative sets.

## Conclusion

Our results demonstrated that SII, NLR were all with great values predicting the grades of GS and GSU, SII seemed be a more favorable tool for it has the largest AUC area compared with NLR; in addition, the combination of SII or NLR with tPSA had great value for predicting GS and GSU and seemed be more favorable when compared with these three factors alone, since the AUC area of combination is much higher. Furthermore, SII, NLR were all useful biomarkers for predicting the grade of GS and detecting the GSU among localized PCa patients with biopsy GS ≤ 6. All these factors might take great values in helping determine the real GS before RP surgery and making decisions on the therapy strategies. Furthermore, as an important and actual method in predicting PCa (36), but the role of MRI in predicting the grades of GS and GSU is still controversial, in the future, a study should be designed to detect the role of SII or NLR combined with the parameter of MRI for predicting the real GS before RP surgery.

## Data availability statement

The raw data supporting the conclusions of this article will be made available by the authors, without undue reservation.

## Ethics statement

The studies involving human participants were reviewed and approved by Institutional Review Board of Peking University Cancer Hospital & Institution (protocol code 2020KT30). Written informed consent for participation was not required for this study in accordance with the national legislation and the institutional requirements.

## Author contributions

PD and SW designed the study. SW, YJ and JM made the same contribution in this study as the first co-author. SW, YJ, JM, YC, XY, ZY, PD and YY performed the study and analyzed the data. PD, SW, YJ and JM wrote the manuscript draft and revised the manuscript. All authors contributed to the article and approved the submitted version.

## References

[1] Prostate cancer estimated incidence, and prevalence worldwide in 2012. Available at: http://globocan.iarc.fr/old/FactSheets/Cancers/Prostate-new.asp (Accessed October 4, 2016).

[2] Kasivisvanathan V Rannikko AS Borghi M Panebianco V Mynderse LA Varrala MH. MRI-Targeted or standard biopsy for prostate cancer diagnosis. N Engl J Med (2018) 378:1767–77. doi: 10.1056/NEJMoa1801993 PMC9084630 29552975

[3] Schreiber D Chhabra A Rinner J Weedon J Schwartz D. A population-based study of men with low-volume, low-risk prostate cancer: Does African American race predict for more aggressive disease? Clin Genitourin Cancer (2015) 13:e259–264. doi: 10.1016/j.clgc.2015.02.006 25777681

[4] Vasto S Carruba G Candore G Itaiano E Di Bona D Caruso C. Inflammation and prostate cancer. Future Oncol (2008) 5:637–45. doi: 10.2217/14796694.4.5.637 18922121

[5] Nakai Y Nonomura N. Inflammation and prostate carcinogenesis. Int J Urol (2013) 20:150–60. doi: 10.1111/j.1442-2042.2012.03101.x 22852773

[6] Kawahara T Fukui S Sakanmaki K Ito Y Ito H Kobayashi N. Neutrophil-to-lymphocyte ratio predicts prostatic carcinoma in men undergoing needle biopsy. Oncotarget (2015) 6:32169–76. doi: 10.18632/oncotarget.5081 PMC4741667 26359354

[7] Gokce MI Tangal S Hamidi N Suer E Ibis MA Beduk Y. Role of neutrophil to lymphocyte ratio in prediction of Gleason score upgrading and disease upstaging in low-risk prostate cancer patients eligible for active surveillance. Can Urol Assoc (2016) 10:11–2. doi: 10.5489/cuaj.3550 PMC5234405 28096923

[8] Rulando M Siregar GP Warli SM. Correlation between neutrophil-to-lymphocyte ratio with Gleason score in patients with prostate cancer at Adam malik hospital medan. Urol Ann (2021) 13:53–5. doi: 10.4103/UA.UA_1_20 PMC8052910 33897165

[9] Chung MS Lee SH Lee DH Chung BH. Is small prostate volume a predictor of Gleason score upgrading after radical prostatectomy? Yonsei Med J (2013) 54:902–6. doi: 10.3349/ymj.2013.54.4.902 PMC3663210 23709424

[10] Imamoto T Utsumi T Takano M Komaru A Fukasawa S Suyama T. Development and external validation of a nomogram predicting the probability of significant gleason sum upgrading among Japanese patients with localized prostate cancer. Prostate Cancer (2011) 754382. doi: 10.1155/2011/754382 22110999 PMC3216057

[11] Hu B Yang XR Xu Y Sun YF Sun C Guo W. Systemic immune inflammation index predicts prognosis of patients after curative resection for hepatocellular carcinoma. Clin Cancer Res (2014) 20:6212–22. doi: 10.1158/1078-0432.CCR-14-0442 25271081

[12] Lolli C Basso U Derosa L Scarpi E Sava T Santoni M. Systemic immune inflammation index predicts the clinical outcome in patients with metastatic renal cell cancer treated with sunitinib. Oncotrarge (2014) 7:54564–71. doi: 10.18632/oncotarget.10515 PMC5342364 27409344

[13] Passardi A Scarpi E Cavanna L Dall’Agata M Tassinari D Leo S. Inflammatory indexes as predictors of prognosis and bevacizumab efficacy in patients with metastatic colorectal cancer. Oncotarget (2016) 7:33210–9. doi: 10.18632/oncotarget.8901 PMC5078087 27120807

[14] Lolli C Caffo O Scarpi E Aieta M Conteduca V Maines F. Systemic immune inflammation index predicts the clinical outcome in patients with mCRPC treated with abiraterone. Front Pharmacol (2016) 13:376. doi: 10.3389/fphar.2016.00376 PMC5062111 27790145

[15] Walsh PC Retik AB Vaughan ED Harrison JH Gittes RF Perlmutter AD. Anatomic radical retropubic prostatectomy; in campbell’s urology (ed 8). Philadephia (2002) 4:3107–29. doi: 10.1016/j.sxmr.2017.11.003

[16] Epstein JI Egevad L Amin MB Delahunt B Srigley JR Humphrey PA. The 2014 international society of urological pathology (ISUP) consensus conference on Gleason grading of prostatic carcinoma: definition of grading patterns and proposal for a new grading system. Am J Surg Pathol (2016) 40:244–52. doi: 10.1097/PAS.0000000000000530 26492179

[17] Frederick L Page DL Fleming ID Fritz AG Greene FL. AJCC cancer staging manual. New York, NY, USA: Springer Science & Business Media (2002).

[18] Chovanec N Cierna Z Miskovska V Machalekova K Kalavska K Rejlekova K. Systemic immune-inflammation index is prognostic in testicular germ cell tumors with PD-L1 expressing tumor infiltrating lymphocytes. J Clin Oncol (2017) 35:e16042. doi: 10.1200/JCO.2017.35.15_suppl.e16042 PMC5400624 28423520

[19] Eggener SE Scardino PT Walsh PC Han M Partin AW Trock BJ. Predicting 15-year prostate cancer specific mortality after radical prostatectomy. J Urol. (2011) 185:869–75. doi: 10.1016/j.juro.2010.10.057 PMC4058776 21239008

[20] Wang S Du P Cao Y Yang X Yang Y. Tumor biological feature and its association with positive surgical margins and apical margins after radical prostatectomy in non-metastasis prostate cancer. Curr Oncol (2021) 28:1528–36. doi: 10.3390/curroncol28020144 PMC8167593 33924669

[21] Moreno D Sanchez L Migallon Raya L Rueda JA Gomez O. Inflammatory markers as prognostic factors in metastatic castration resistant prostate cancer. Actas Urol Esp (2020) 10:692–700. doi: 10.1016/j.acuro.2020.08.001 33010988

[22] Sved PD Gomez P Manoharan M Kim SS Soloway MS. Limitation of biopsy gleason grade: implications for counseling patients with biopsy gleason score 6 prostate cancer. J Urol (2004) 172:98–102. doi: 10.1097/01.ju.0000132135.18093.d6 15201746

[23] Cookson MS Fleshner NE Soloway SM Fair WR. Correlation between gleason score of needle biopsy and radical prostatectomy specimen: accuracy and clinical implications. J Urol. (1997) 157:559–62. doi: 10.1016/S0022-5347(01)65201-7 8996356

[24] Wang H Gu L Wu Y Feng D Duan J Wang XC. The values of neutrophil-lymphocyte ratio and/or prostate specific antigen in discriminating real Gleason Score≥7 prostate cancer from group of biopsy-based gleason score≤6. BMC cancer. (2017) 17:629–37. doi: 10.1186/s12885-017-3614-9 PMC5586011 28874127

[25] Cary KC Cooperberg MR. Biomarkers in prostate cancer surveillance and screening: Past, present, and future. Ther Adv Urol (2013) 5:318–29. doi: 10.1177/1756287213495915 PMC3825107 24294290

[26] Kwang HK Sey KL Tae YS Lee JY Chung BH Rha KH. Upgrading of Gleason score and prostate volume: a clinicopathological analysis. BJU Int (2013) 111:1310–6. doi: 10.1111/j.1464-410X.2013.11799.x 23452115

[27] Massanova M Robertson S Barone B Dutto L Caputo VF Bhatt JR. The comparison of imaging and clinical methods to estimate prostate volume: a single center retrospective. Urol Int (2021) 105:804–10. doi: 10.1159/000516681 34247169

[28] Sun Z Ju Y Han F Sun X Wang F. Clinical implications of pretreatment inflammatory biomarkers as independent prognostic indicators in prostate cancer. J Clin Lab Anal (2018) 32:e22277. doi: 10.1002/jcla.22277 28605139 PMC6816827

[29] Van Soest RJ Templeton AJ Vera-Badillo FE Mercier F Sonpavde G Amir E. Neurtophil-to-lymphocyte ratio as a prognostic biomarker for men with metastatic castration-resistant prostate cancer receiving fist line chemotherapy: data from two randomized phase 3 trials. Ann Oncol (2015) 26:743–9. doi: 10.1093/annonc/mdu569 25515657

[30] Jang WS Cho KS Kim MS Yoon CY Kang DH Kang YK. The prognostic significance of postoperative neutrophil-to-lymphocyte ratio after radical prostatectomy for localized prostate cancer. Oncotarget (2017) 8:11778–87. doi: 10.18632/oncotarget.14349 PMC5355303 28052031

[31] Wang S Ji Y Chen Y Du P Cao YD Yang X. The values of systemic immune-inflammation index and neutrophil-lymphocyte ratio in the localized prostate cancer and benign prostate hyperplasia: a retrospective clinical study. Front Oncol (2022) 11:812319. doi: 10.3389/fonc.2021.812319 35047413 PMC8763322

[32] Wang S Yang X Yu Z Du P Sheng XN Cao YD. The values of systemic immune-inflammation index and neutrophil-lymphocyte ratio in predicting biochemical recurrence in patients with localized prostate cancer after radical prostatectomy. Front Oncol (2022) 12:907625. doi: 10.3389/fonc.2022.907625 35719913 PMC9200963

[33] Wang K Diao F Ye Z Zhang X Zhai E Ren H. Prognostic value of systemic immune inflammation index in patients with gastric cancer. Clin J Cancer. (2017) 36:75. doi: 10.1186/s40880-017-0243-2 PMC5596912 28899420

[34] Man Y Chen Y. Systemic immune-inflammation index, serum albumin, and fibrinogen impact prognosis in castration resistant prostate cancer patients treated with first line docetaxel. Int Urol Nephrol. (2019) 12:2189–99. doi: 10.1007/s11255-019-02265-4 31456101

[35] Rajwa P Schuettfort VM Andrea DD Quhal F Mori K Katayama S. Impact of systemic immune inflammation index on oncologic outcomes in patients treated with radical prostatectomy for clinically nonmetastatic prostate cancer. Urol Oncol (2021) 39:785.e19–785.e27. doi: 10.1016/j.urolonc.2021.05.002 34116934

[36] Ferro M Crocetto F Bruzzese D Massimo I Ferdinando F Nicola L. Prostate health index and multiparametric MRI: Partners in crime fighting overdiagnosis and overtreatment in prostate cancer. Cancers (2021) 13:4723. doi: 10.3390/cancers13184723 34572950 PMC8466029

